# Electricity consumption in Finland influenced by climate effects of energetic particle precipitation

**DOI:** 10.1038/s41598-023-47605-8

**Published:** 2023-11-23

**Authors:** Veera Juntunen, Timo Asikainen

**Affiliations:** Space Physics and Astronomy Research Unit, University of Oulu, Oulu, Finland

**Keywords:** Space physics, Atmospheric science

## Abstract

It is known that electricity consumption in many cold Northern countries depends greatly on prevailing outdoor temperatures especially during the winter season. On the other hand, recent research has demonstrated that solar wind driven energetic particle precipitation from space into the polar atmosphere can influence the stratospheric polar vortex and tropospheric weather patterns during winter. These changes are significant, e.g., in Northern Europe, especially in Finland. In this study we demonstrate that geomagnetic activity, as a proxy of energetic particle precipitation, significantly influences Finland’s average temperature and total wintertime electricity consumption in Finland. This influence is only seen when the prevailing equatorial stratospheric winds, so called QBO winds, are easterly. The results demonstrate a previously unrecognized societal influence of space weather, and imply that long-term energy consumption forecasts could potentially be improved by considering long-term space weather predictions.

## Introduction

Prevailing weather conditions, especially outdoor temperatures are known to have a pronounced effect on electricity consumption in different European countries^1–3^. During wintertime a large fraction of total electricity consumption is used for heating in countries that have cold winter temperatures, including Finland^1^. Therefore, any factor influencing the wintertime weather conditions directly affects the total electricity consumption as well. For example, it has been shown that the North Atlantic Oscillation (NAO), the dominant air pressure pattern over the North Atlantic which largely governs the type of wintertime weather in Europe, influences, e.g., the energy consumption in Norway^4^, the electricity markets in Ireland^5^, and the energy penetration rate in Europe^6^. While much of the wintertime weather variability in the Northern Hemisphere arises from internal tropospheric variability, it is also known that a significant influence to the this tropospheric variability is exerted from the stratosphere^7^. During wintertime the polar stratosphere is characterized by the polar vortex, a strong westerly wind flow that circulates the cold and dark winter polar region in high-latitude stratosphere. Variations of the polar vortex have been shown to project on the so called Northern Annular Mode (NAM)^8^ and North Atlantic Oscillation (NAO)^9^ modes of air pressure variability throughout the stratosphere down to the troposphere and ground level^7,10^.

An interesting aspect to all this is brought by the fact that varying solar activity and corresponding changes in space weather have been shown to influence the Earth’s middle atmosphere and the climate system (e.g.^11–15^). Particularly, it has been noted that energetic electron precipitation (EEP) from near-Earth space, mainly driven by fast solar wind streams from the solar corona^16^, influence the polar vortex and wintertime climate conditions on the Northern Hemisphere^17–19^. This influence is exerted by chemical destruction of stratospheric ozone by odd hydrogen (HOx) and nitrogen oxides (NOx), which are created when energetic particles ionize the upper atmosphere^20–22^. The NOx compounds are especially important because in the absence of sunlight they can remain in the polar atmosphere for long time periods and be transported from the upper atmosphere down to the mesosphere and stratosphere where they then catalytically destroy ozone^23–25^. Since ozone is an important regulator of stratospheric temperature, variations in its concentrations change stratospheric heating rates and temperatures^26,27^. In dark early and mid winter ozone depletion decreases radiative cooling rates and leads to warming of the mesosphere and upper stratosphere, while in late winter when sunlight starts to return to the polar region the ozone loss leads to decreased radiative heating rate, which results in cooling of the stratosphere^22,28^. These thermal changes ultimately lead to enhancement of the polar vortex in accordance with the thermal wind shear balance.

Recent research has demonstrated that the above described influence of EEP on the polar vortex is dependent on the distribution of planetary waves, large scale north-south undulations of wind streams, which are caused by fluctuations of tropospheric air pressure centers^19,29^. While these waves can propagate to the stratosphere and influence the zonal winds there, their propagation is also influenced by the zonal winds thereby setting up a back and forth feedback known as the wave-mean-flow interaction^30^. An important factor affecting the guiding of planetary waves to the polar stratosphere is the Quasi-Biennial Oscillation (QBO) of equatorial stratospheric zonal winds^31^. The direction of the wind changes around every 14 months between easterly and westerly. During easterly QBO winds (negative phase) the planetary waves are guided more effectively from the mid-latitudes to the polar region^32^. There they have been suggested to act as an amplifier of the EEP-induced changes of the polar vortex. Consequently the EEP influence on the polar vortex is preferentially seen during the easterly phase of the QBO (e.g.^19,29,33,34^). While a high level of EEP enhances the polar vortex a low EEP level has been shown to make the vortex prone to breaking leading to a Sudden Stratospheric Warming^35,36^.

As the strength of the stratospheric polar vortex influences the surface temperatures, the polar vortex also works as a link transmitting the EEP effects to the surface level^17,19^. As mentioned earlier, these changes largely project to the NAM/NAO modes of climate variability and, e.g., in Northern Europe/Scandinavia, especially in Finland, lead to warm and wet (cold and dry) winter weather when the EEP level is high (low) in easterly QBO phase^17,33^.

Based on this premise, this study now considers for the first time how the EEP related climate variability influences the total electricity consumption in Finland, for which detailed long-term energy statistics since early 1990’s are available. We first show that, apart from long-term trends, Finland’s total electricity consumption correlates extremely well with Finland’s average temperature. We then demonstrate that geomagnetic activity (an indirect proxy for EEP) influences the surface geopotential patterns and thereby affects Finland’s wintertime temperatures. Finally, we show that this connection projects into a significant correlation between geomagnetic activity and total electricity consumption in Finland when the phase of the QBO easterly.

## Results

### Reconstruction of the total electricity consumption by using surface temperature data

**Figure 1:**
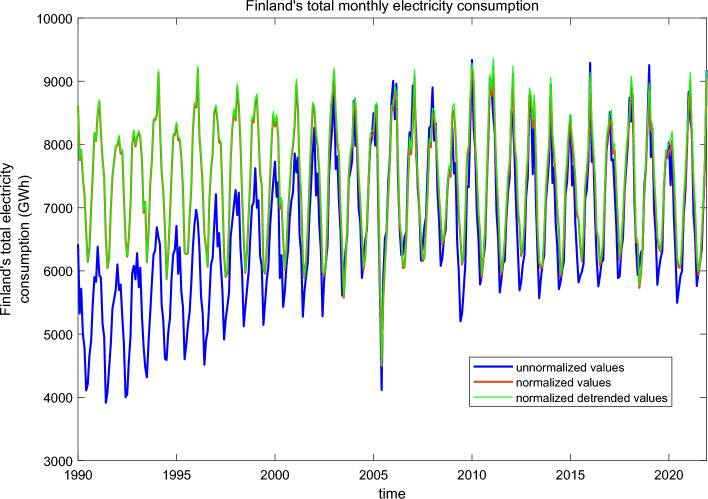
Finland’s total monthly electricity consumption from 1990 to 2021 (blue) and normalized values (red). The green curve shows the normalized detrended monthly electricity consumption values (linear trend removed from each month separately) used in the analysis.

Figure 1 shows monthly averages of Finland’s total electricity consumption from 1990 to 2021 obtained from the archives of Finnish Energy (see “Methods” section). The evolution of electricity consumption is dominated by a non-linear trend increasing from 1990 to about 2008 after which it has stayed relatively constant. Another persistent feature is the strong seasonal variation, which peaks in winter. Evidently, there is also year-to-year variability in electricity consumption, which is not related to weather variations but to changes in industry and overall energy consumption. In this study we concentrate only on the part of the inter-annual variations in electricity consumption, which can be attributed to variability of Finland’s average surface temperature obtained from the ERA-5 re-analysis dataset (see “Methods” section). Extracting the temperature dependent part of electricity consumption variability involves normalizing the electricity consumption with temperature and removing the long-term trends not associated to temperature changes (see “Methods” section). Figure 1 shows the normalized electricity consumption values with a red curve.

**Figure 2:**
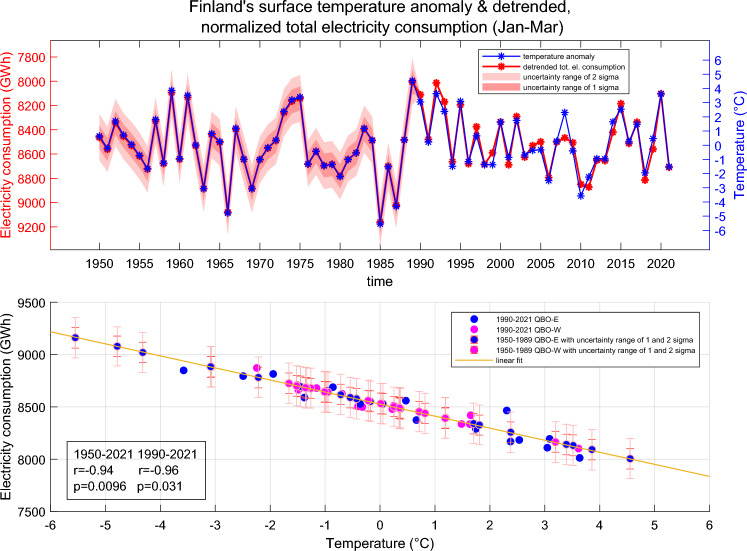
The Jan–Mar (JFM) averages of normalized detrended total electricity consumption and Finland’s surface temperature anomaly (JFM) in 1950–2021. The scatter plot shows QBO-E winters in blue and QBO-W winters in pink. The QBO has been taken from July preceding the winter season. The red shading in the time series plot and red bars in the scatter plot indicate the uncertainty range of 1 and 2 sigma for the reconstructed electricity consumption values.

After the normalization procedures we find that all calendar months and different years are systematically similarly related to Finland’s monthly average temperature, and the correlation of this relation is very high (cc = − 0.98, p $< 10^{-279}$). The good correlation between temperature and normalized electricity consumption allows us to calculate by linear regression a representative estimate for the normalized electricity consumption for 1950–1989 using Finland’s monthly temperatures as proxies (see “Methods” section). Figure 2 shows the Jan–Mar (JFM) averages of Finland’s average temperature and the normalized total electricity consumption in 1950–2021 both as time series (top) and a scatter plot (bottom). The interval from 1950 to 1989 consists of the reconstructed electricity consumption data and the red shading indicates the one and two standard deviation (sigma) uncertainty ranges of the reconstruction. From Fig. 2 one can clearly see how average temperatures (right side axis) translate to electricity consumption values (left side axis).

### Influence of geomagnetic activity on surface temperature

Earlier studies have already established the influence of EEP on large-scale ground temperature and sea level pressure patterns in the Northern Hemisphere during winter (e.g.^14,17,33,34^). For example, Maliniemi et al.^17^ showed that the EEP influence projects to a NAM like pattern predominantly during easterly QBO phase and is associated to warming in northern Eurasia, where Finland is also located. Let us now investigate more closely how Finland’s average temperature in winter is associated to global patterns of geopotential height in the Northern Hemisphere and how those patterns are connected to geomagnetic activity (aa index), which is here used as a proxy for energetic electron precipitation.

**Figure 3:**
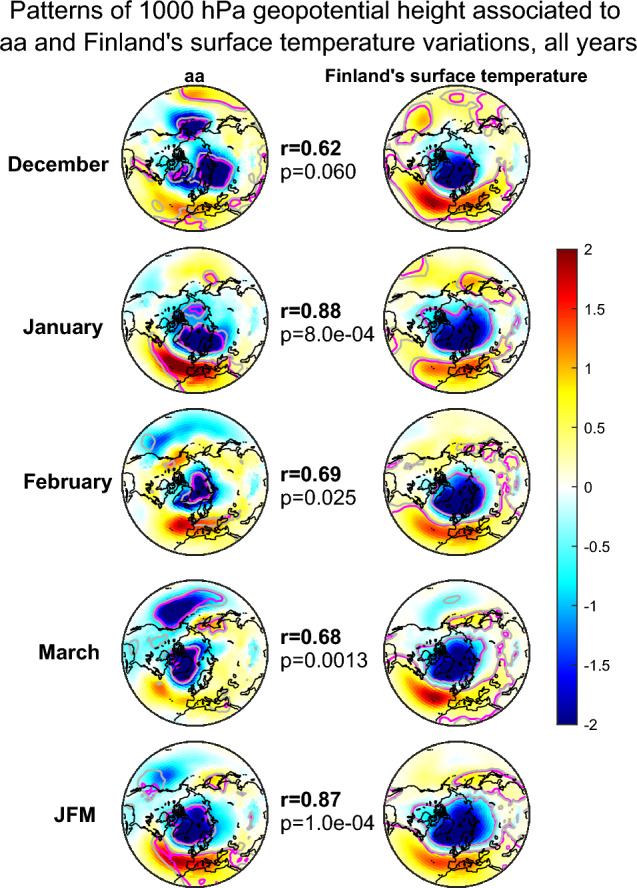
Patterns of maximum covariance between 1000 hPa geopotential height and detrended aa on the left and Finland’s surface temperature anomaly on the right. Different rows correspond to different winter months and the Jan–March (JFM) average. The correlations and p-values indicate the correlation and statistical significance between aa and temperature associated maximum covariance patterns. The patterns shown here were computed for 1950–2021 excluding winters 84/85 and 03/04. The grey (pink) contours indicate 90% (95%) significance levels. The colorbar represents dimensionless MCA pattern loadings scaled by dividing with the standard deviation of all loading values of the same map.

**Figure 4:**
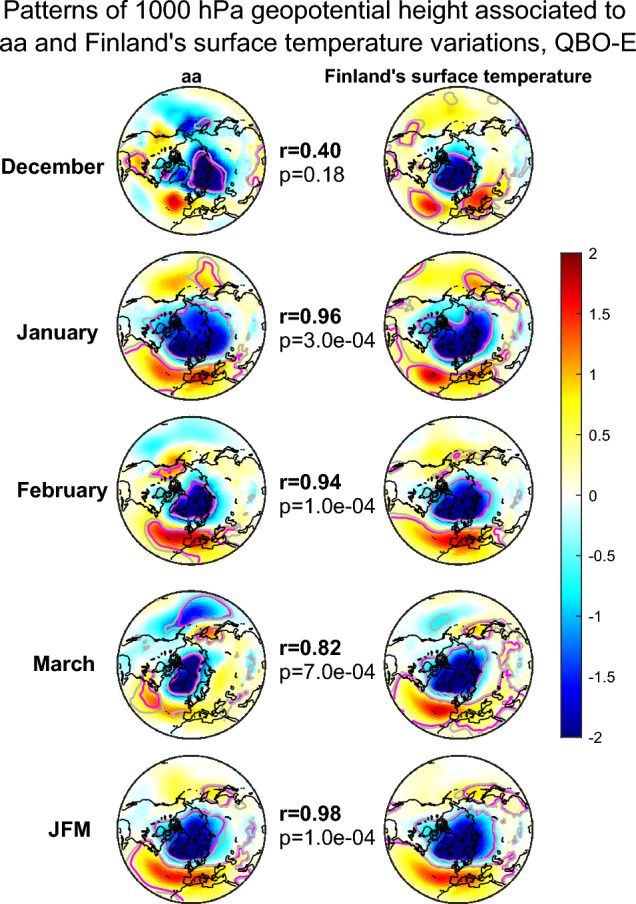
Patterns of maximum covariance between 1000 hPa geopotential height and detrended aa on the left and Finland’s surface temperature anomaly on the right for the QBO-E winters. Different rows correspond to different winter months and the Jan–March (JFM) average. The correlations and p-values indicate the correlation and statistical significance between aa and temperature associated maximum covariance patterns. The patterns shown here were computed for 1950–2021 excluding winters 84/85 and 03/04. The grey (pink) contours indicate 90% (95%) significance levels. The colorbar represents dimensionless MCA pattern loadings scaled by dividing with the standard deviation of all loading values of the same map.

**Figure 5:**
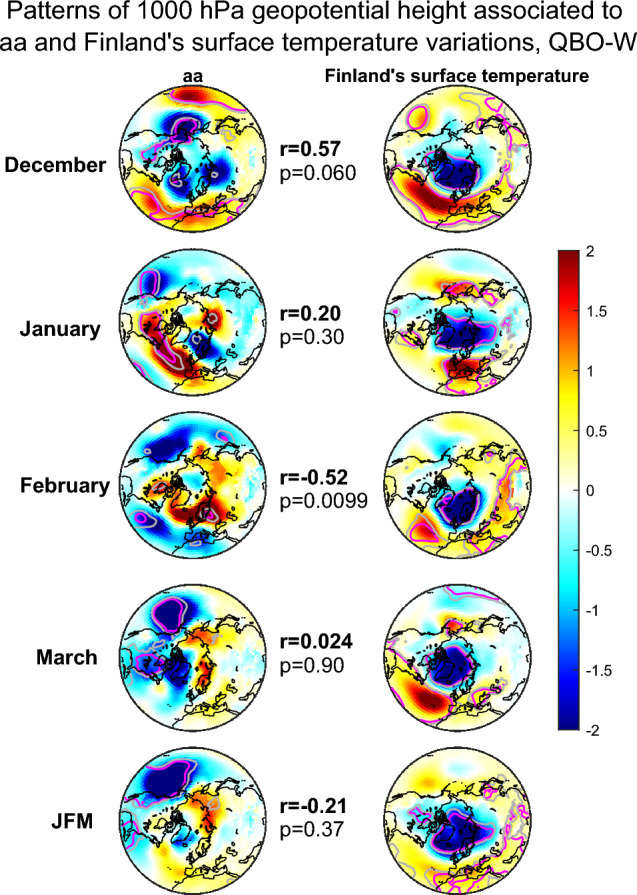
Patterns of maximum covariance between 1000 hPa geopotential height and detrended aa on the left and Finland’s surface temperature anomaly on the right for the QBO-W winters. Different rows correspond to different winter months and the Jan–March (JFM) average. The correlations and p-values indicate the correlation and statistical significance between aa and temperature associated maximum covariance patterns. The patterns shown here were computed for 1950–2021 excluding winters 84/85 and 03/04. The grey (pink) contours indicate 90% (95%) significance levels.The colorbar represents dimensionless MCA pattern loadings scaled by dividing with the standard deviation of all loading values of the same map.

Figures 3, 4 and 5 show the patterns of 1000 hPa geopotential height, which are associated to variation of aa index (left side panels) and Finland’s average temperature (right side panels) in 1950–2021 separately for all winters (Fig. 3), QBO-E winters (Fig. 4) and QBO-W winters (Fig. 5). We deseasonalize the QBO (see “Methods” section) and employ here a 6 month lag to the QBO so that it is taken from July preceding the winter season. Earlier studies on the EEP effect have found the 6 month lagged QBO to influence the EEP effect more strongly than other lags^19^. The different rows of Figs. 3, 4 and 5 represent different winter months with the bottom row showing the JFM average. These patterns were obtained by maximum covariance analysis and projecting them on the monthly geopotential height data produces a time series, which maximally covaries with either aa index or Finland’s average temperature time series (see “Methods” section). It is important to note that for February, March and JFM average the aa index was taken from preceding January. A similar time lag for the EEP (aa) influence has been found in many earlier studies (e.g.,^19,33^). We have also excluded from the analysis winters 1984/1985 and 2003/2004, which experienced large sudden stratospheric warming events breaking the polar vortex for a long time. These two winters have been found exceptional in several earlier studies and they are strong outliers, which greatly dilute the estimated EEP (and aa) related variability in the polar vortex and ground climate^17,19,37^. Figures 3 and 4 also show the Pearson correlation coefficients and p-values between aa and Finland’s temperature related patterns in the middle (see “Methods” section) and they indicate how similar the two patterns are (however, they do not represent the correlation between aa and Finland’s temperature time series).

For all winters (Fig. 3) the geomagnetic activity in Dec–Feb months is associated to a pattern that resembles the NAM/NAO patterns. Generally, the NAM pattern has a negative response over the pole surrounded by patches of positive responses over northern Atlantic and Pacific oceans. The NAO pattern lacks the positive response over Pacific and thus resembles the Atlantic sector of the NAM pattern. In all winter months Finland’s temperature is maximally associated with a NAM/NAO like pattern. Especially in January and February as well as in JFM averages the geomagnetic activity is associated to a NAM/NAO like pattern, by which Finland’s temperature is predominantly influenced. The correlation between the two patterns, e.g., for the JFM averages is high and statistically significant ($r = 0.87$, $p = 1.0 \times 10^{-4}$). Figure 4 shows the same patterns for QBO-E phase. In accordance with earlier studies, the aa-related response is now more systematically NAM/NAO-like throughout all winter months, except December. The geopotential pattern associated to Finland’s average temperatures is not significantly different from Fig. 3. However, the correlations between the aa patterns and Finland’s average temperature related patterns are now much stronger and more systematic for Jan–Mar months. Especially the JFM average patterns attain a very strong and highly significant correlation of $r=0.98$ ($p=1.0 \times 10^{-4}$). In QBO-W phase (Fig. 5) the geopotential patterns associated to Finland’s temperature are similar as in QBO-E, but the patterns associated to aa index are very different resembling almost a negative NAM/NAO pattern. The correlation between the aa and temperature related geopotential patterns is positive in December, rather weak and insignificant in January, moderately negative in February and zero in March. The correlation for the JFM average remains negative ($r = - 0.21$, $p=0.37$), and reflects the correlation in February.

The results in Figs. 4 and 5 indicate that especially in QBO-E phase geomagnetic activity drives a NAM/NAO-like pattern and at the same time a NAM/NAO-like pattern strongly influences Finland’s average temperatures. While Finland’s average temperature responds to a similar NAM/NAO-like pattern also in QBO-W the geomagnetic activity does not systematically produce a positive NAM/NAO-like pattern then. If any, the pattern bears more resemblance to a negative NAM/NAO. It is, therefore, expected that the geomagnetic activity influences Finland’s temperature and electricity consumption predominantly during QBO-E phase. We shall investigate this next.

### Relationship between the geomagnetic activity and Finland’s wintertime total electricity consumption

Table 1 shows the correlation coefficients and corresponding p-values between January’s aa-index and the JFM average of the total electricity consumption in Finland separately for all winters, for QBO-E winters and for QBO-W winters. The statistical significance of the correlations was estimated with a Monte–Carlo resampling method which also estimates the likelihood of obtaining the observed results by aliasing, when the data is irregularly sampled by the QBO phase (see “Methods” section). We use here the January’s aa-index as in Figs. 3, 4 and 5 since it produces best correlation with the JFM average of the total electricity consumption and corresponds to the lagged EEP influence through downwelling of NOx inside the polar vortex as discussed earlier above. Table 1 shows that for the direct measurements in 1990–2021, when winter 2003/2004 has been excluded, the correlation for all winters is moderately negative but not statistically significant ($r = - 0.42$, $p=0.095 > 0.05$). For QBO-E winters the correlation is strongly and significantly negative ($r = - 0.83$, $p=4.0 \times 10^{-4}$) and for the QBO-W winters near zero and statistically insignificant ($p=0.99$). The Table 1 also shows the correlations obtained if the outlier winter of 2003/2004, which occurred in QBO-E phase, is retained in the data. One can see that including this 1 year dramatically decreases the magnitude of the correlation in QBO-E phase from − 0.83 to − 0.57, but the correlation remains statistically significant.

**Table 1:** Pearson correlation coefficients and corresponding p-values between detrended aa-index (Jan) and normalized detrended Finland’s total electricity consumption (JFM).

JFM	All winters	QBO-E	QBO-W
1950–2021 (Winters 84/85 and 03/04 removed)	r = − 0.42 (p=0.0081)	r = − 0.71 (p=2.0 x 10^-4^)	r = 0.019 (p=0.91)
1950–2021 (Winter 03/04 removed)	r = − 0.36 (p=0.0045)	r = − 0.60 (p=8.0 x 10^-4^)	r = 0.019 (p=0.91)
1950–2021 (All winters included)	r = − 0.33 (p=0.0070)	r = − 0.51 (p=0.0020)	r = 0.019 (p=0.91)
1990–2021 (Winter 03/04 removed)	r = − 0.42 (p=0.095)	r = − 0.83 (p=4.0 x 10^-4^)	r = − 0.0043 (p=0.99)
1990–2021 (All winters included)	r = − 0.35 (p=0.12)	r = − 0.57 (p=0.042)	r = − 0.0043 (p=0.99)

Correlations and p-values for 1950–2021 and p-values for 1990–2021 were obtained from Monte Carlo simulations.

For the longer time period 1950–2021, partly consisting of the reconstructed electricity consumption values, the correlations are a bit smaller, but statistically much more significant due to the increased number of data points compared to the 1990–2021 period. It is important to note that the statistical uncertainty range of the electricity consumption reconstruction in 1950–1989 was taken into account when evaluating its correlation with aa-index (see “Methods” section). The correlations shown in Table 1 also imply that the aa influences the electricity consumption predominantly during QBO-E phase, while no statistically significant connection is seen during QBO-W.

For the 1950–2021 time period another particularly influential outlier, winter 1984/1985, exists in the data. This winter has also been identified as an outlier in earlier studies^17^. Excluding also the 1984/1985 winter significantly improves the correlation in QBO-E phase to − 0.71 from − 0.51 (if all data is included) or from − 0.60 (if only 2003/2004 is neglected).

**Figure 6:**
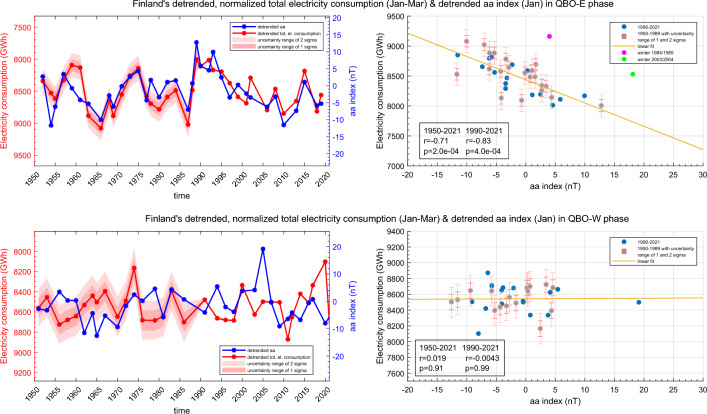
The normalized and detrended total electricity consumption (JFM average) and the detrended aa-index (Jan) during 1950–2021 for QBO-E winters (top row) and for QBO-W winters (bottom row). The QBO was taken from July preceding the winter season. The grey points in the scatter plots indicate reconstructed electricity consumption values. The error limits indicate uncertainty range of 1 and 2 standard deviations (sigmas) for the reconstructed electricity consumption values. The correlation coefficients indicated in the scatter plots have been computed by excluding the two outlier winters of 1984/1985 and 2003/2004 (pink and green).

The connection between the geomagnetic activity and electricity consumption is further investigated in Fig. 6 which shows the time series and the corresponding scatter plots of the January aa-index and the variability of JFM-averaged normalized total electricity consumption of Finland (note the inverted y-axis) separately for the two QBO phases. The upper right hand scatter plot of Fig. 6 also highlights the two outlying winters of 1984/1985 and 2003/2004.

We also considered each winter month’s electricity consumption (December, January, February and March) separately and compared them with the aa index. The correlations between the two are shown in Table 2. In this comparison the aa index was taken from the previous or the same month (as shown in Table 2) as electricity consumption, depending on which month produces the best correlation in QBO-E phase. For December the best correlation was obtained with November aa, although overall the correlations in both QBO phases remain a somewhat low and insignificant for the longer period 1950–2021. For the shorter period 1990–2021 a marginally significant correlation of − 0.47 was obtained for QBO-E phase. For January and February the electricity consumption correlates best with aa index from January. The correlations for Jan and Feb in QBO-E are − 0.60 and − 0.63 for 1950–2021 period and − 0.76 and − 0.68 for 1990–2021 period in QBO-E, and in all cases statistically highly significant. In QBO-W phase the correlations for these months are weak and insignificant. For March the best correlation is found when using aa index from February. In QBO-E phase the correlations are − 0.55 for 1950–2021 period and − 0.63 for the 1990–2021 period and in both cases highly significant. Therefore, generally, the correlations are a bit better for shorter time period 1990–2021, but statistically much more significant for the longer time period 1950–2021 containing more data points. We also note that the correlations for the individual winter months are somewhat lower than for the JFM average electricity consumption. This is evidently, because taking the average of three winter months reduces the variance of the contribution that comes from factors not related to aa variations.

**Table 2:** Pearson correlation coefficients and corresponding p-values between Finland’s normalized detrended total electricity consumption and detrended aa-index for 1950–2021 and 1990–2021.

Month	All winters	QBO-E	QBO-W
December 1950–2021 (aa Nov)	r = − 0.084 (p=0.49)	r = − 0.24 (p=0.19)	r = 0.12 (p=0.52)
December 1990–2021 (aa Nov)	r = − 0.040 (p=0.90)	r = − 0.47 (p=0.077)	r = 0.32 (p=0.23)
January 1950–2021 (aa Jan)	r = − 0.34 (p=0.0079)	r = − 0.60 (p = 2.0 x 10^-4^)	r = − 0.019 (p=0.92)
January 1990–2021 (aa Jan)	r = − 0.42 (p=0.034)	r = − 0.76 (p=0.0017)	r = − 0.17 (p=0.55)
February 1950–2021 (aa Jan)	r = − 0.31 (p=0.011)	r = − 0.63 (p=6.0 x 10^-4^)	r = 0.028 (p=0.87)
February 1990–2021 (aa Jan)	r = − 0.28 (p=0.19)	r = − 0.68 (p=0.016)	r = − 0.031 (p=0.92)
March 1950–2021 (aa Feb)	r = − 0.29 (p=0.011)	r = − 0.55 (p=9.0 x 10^-4^)	r = 0.028 (p=0.87)
March 1990–2021 (aa Feb)	r = − 0.40 (p=0.031)	r = − 0.63 (p=0.011)	r = − 0.036 (p=0.90)

The month indicates the month of the total electricity consumption. The QBO has been taken from July preceding the winter season and the aa from previous or same month as indicated in the table. Here winters 1984/1985 and 2003/2004 have been excluded from all correlations. Correlations and p-values for 1950–2021 and p-values for 1990–2021 were obtained from Monte Carlo simulations.

The correlation between aa and the electricity consumption found here is due to the influence of energetic electron precipitation on the Finland’s average temperature. The correlations between aa and temperature are not shown here explicitly as they are not significantly different from those between aa and electricity consumption (due to the strong correlation between temperature and normalized electricity consumption shown above).

## Discussion

Past research has demonstrated that energetic electron precipitation into the atmosphere can influence the wintertime polar vortex when the QBO phase is easterly (e.g.,^19,33,35^). In westerly QBO phase this influence is much weaker, likely because the EEP influence on polar vortex has been shown to depend on planetary waves^29,38^, which are preferentially guided into the polar stratosphere during QBO-E phase^32^. The EEP-related influences can even be seen as statistically significant variations of ground temperatures in the Northern Hemisphere. Here we also demonstrated how the aa index of geomagnetic activity (proxy for energetic electron precipitation) projects its influence on a NAM-type pattern of 1000 hPa geopotential height (Fig. 4) in the QBO-E phase. On the other hand Finland’s wintertime temperatures were also shown to be dominantly dependent on a similar NAM-type pattern. Based on this the aa-associated variations greatly influence the wintertime (Jan–Mar) temperatures in Finland in QBO-E phase.

Based on this premise we studied here how the influence of geomagnetic activity is seen in the wintertime total electricity consumption in Finland. After carefully extracting the part of electricity consumption attributed to temperature changes we found a statistically significant and rather high correlation between the normalized electricity consumption and aa index in QBO-E winters since 1950–2021, which suggests that more than 50% of the temperature related part of electricity consumption can be explained by variations of geomagnetic activity. In QBO-E phase increased geomagnetic activity (high aa) leads to higher winter temperatures in Finland which in turn reduces the total electricity consumption as less electricity is needed for heating. Correspondingly when geomagnetic activity is decreased (low aa), Finland experiences colder winter temperatures causing increased total electricity consumption.

Figure 6 indicates that the average level of the electricity consumption (normalized to the level of year 2021) is about 8500 GWh per Jan–Mar season. On the other hand the range of variability of the electricity consumption is about 1200 GWh. Based on this the geomagnetic activity associated part of the electricity consumption is about 14% of the average level. This magnitude is not negligible and has two major implications. On one hand, it indicates a significant societal influence of space weather (geomagnetic activity associated particle precipitation into the atmosphere) which has not been previously recognized. On the other hand, the results imply that long-term predictions of electricity consumption for months ahead could potentially be improved by considering long-term forecasts of the space environment and their influence on wintertime ground weather variations (e.g.^36^).

## Methods

### Data

We use the fifth generation of ECMWF’s (European Centre for Medium-range Weather Forecasts) atmospheric reanalysis dataset called ERA5 (https://cds.climate.copernicus.eu) for geopotential height, equatorial stratospheric zonal wind (QBO) and surface temperature data^39^. The dataset has a spatial resolution of $0.25^\circ$ both in latitude and longitude over entire globe in 37 different pressure levels from 1000 to 1 hPa. Here we used data from 1950 to 2021. For this study we computed Finland’s monthly average surface temperature using the ERA-5 dataset as a mean value between latitudes 60$\vphantom{0}^\circ$ N–71$\vphantom{0}^\circ$ N and longitudes 21$\vphantom{0}^\circ$ E–30$\vphantom{0}^\circ$ E. We also used ERA-5 data to determine the QBO as the average zonal wind between latitudes 10$\vphantom{0}^\circ$ S–10$\vphantom{0}^\circ$ N. The QBO was further de-seasonalized by removing from the values of each calendar month the overall average value of the corresponding calendar month.

As a proxy of EEP we used the geomagnetic aa index provided by International Service of Geomagnetic indices (http://isgi.unistra.fr). The aa index has been determined since 1868 and it represents the range of geomagnetic variability measured on ground in 3-h time intervals normalized to $\pm 50 ^\circ$ geomagnetic latitude. The index is based on the data from two stations located in England (presently Hartland) and Australia (presently Canberra). The aa index forms the longest running time series of geomagnetic activity^40^. The aa index and the closely related ap index, containing data from more stations, have often been used as a good proxy for the energetic electron precipitation^16,37,41^.

The Finland’s electricity consumption data was obtained from the Finnish Energy database (https://energia.fi/en/newsroom/publications/monthly_electricity_statistics.html), which contains detailed statistics of electricity production and consumption in weekly and monthly time resolution. In this study we use monthly total electricity consumption data for 1990 to 2021. To extend the time range of monthly electricity consumption data we reconstruct the monthly total electricity consumption dataset for years 1950–1989 by using Finland’s monthly temperature data.

### Processing of electricity consumption data

**Figure 7:**
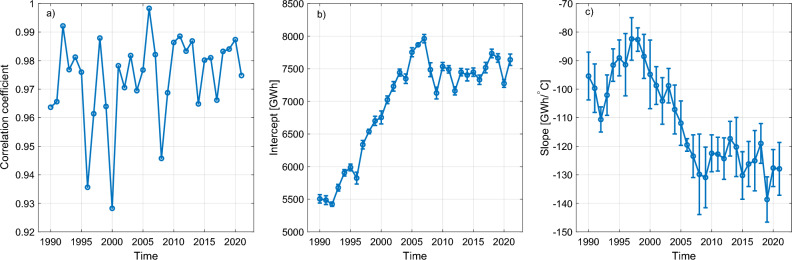
(**a**) The correlation coefficient between monthly total electricity consumption and Finland’s monthly average temperature for different years. (**b,c**) The intercept and slope of the linear fit between electricity consumption and monthly temperature as a function of time.

The electricity consumption data contains the total amount of electricity consumed (GWh) each month. Because different months have different number of days the electricity consumption was first normalized to a constant number of 30 days per month. This is done by multiplying each monthly value by a factor of 30/N, where *N* is the number of days within a month. After this normalization we found that the electricity consumption extremely well linearly correlated with Finland’s monthly average temperature in each year. This is shown in Fig. 7a, which indicates that for all years the correlation exceeds 0.92. However, when fitting a line (electricity consumption vs. temperature) we find that each year has a different intercept and slope as shown in panels b and c of Fig. 7. This is because of the long-term trends in electricity consumption unrelated to temperatures. These are mostly reflected in the intercept (Fig. 7b), which indicates a rising trend until 2007 after which the trend drops slightly and attains a roughly constant level with small year-to-year variations. This trend roughly follows Finland’s overall economical growth indicator^42^.

The long-term changes in how Finland’s electricity consumption responds to outside temperatures is also reflected in the slope of the electricity vs. temperature fit (Fig. 7c). Generally the slopes are always negative indicating that a decrease in temperature leads to increase in electricity consumption. However, the slope seems to systematically decrease towards larger negative values until 2009 after which it attains a somewhat constant level with small year-to-year variations. The decreasing slope especially from 1997 to 2009 indicates that the total electricity consumption became more and more sensitive to outside temperatures. This is likely due to increase in the number of residential and industrial spaces heated with electricity. There has been a steady increase in the overall number of residential and industrial spaces in Finland in the recent decades and between 2005 and 2017 the number of residential and industrial spaces heated with electricity increased from 500,000 to 590,000, i.e. by almost 20%^43^. On the other hand, the slowing in the decrease of the slope after 2009 may be related to increased energy efficiency of heating in residential and industrial sector, e.g., rapid increase of energy efficient air-source heat pumps^44^.

In this study we concentrate on extracting the variability of electricity consumption, which is related to Finland’s temperature variations. Therefore, we first scaled each year separately to have the same linear dependence on temperature as year 2021 (last year of our dataset). For the *i*th year the 12 monthly values were scaled by the following equation

1 $$\begin{aligned} E' = E + (a_{2021} - a_i) + (b_{2021} - b_i)T, \end{aligned}$$

where $E'$ is the scaled electricity consumption, *E* is the unscaled (but normalized to number of days within a month) electricity consumption, $a_{2021}$ and $a_i$ are the intercepts and $b_{2021}$ and $b_i$ the slopes of the linear fits for year 2021 and year *i* respectively. This scaling removes the long-term non-linear trend in the electricity consumption and normalizes all the monthly values in different years to have the same temperature dependence on average. This means that all values with the same temperature correspond on average to same level of electricity consumption, regardless of which calendar month they correspond to.

**Figure 8:**
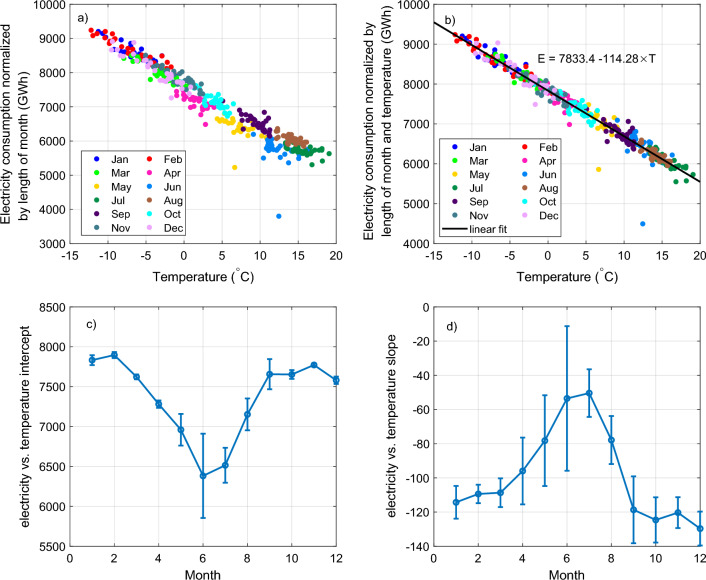
(**a**) Electricity consumption normalized by length of month as a function of Finland’s average monthly temperature. Different colors represent different calendar months. (**b**) Electricity consumption normalized by length of month and temperature as a function of Finland’s average monthly temperature. The panel also shows the linear fit to all points, which is used to reconstruct normalized electricity consumption for years 1950–1989. (**c,d**) Display the intercepts and slopes of linear fits made separately to each calendar month of (**a**), and which were used to obtain the normalized the electricity consumption values shown in (**b**).

However, after this scaling the different calendar months still have differences in the way they separately respond to temperature. This is shown in Fig. 8a, where one sees subtle differences between the different calendar months. Panels c) and d) show the intercepts and slopes of the linear fits made separately to the different calendar months. One can see that the intercept (Fig. 8c) systematically changes over the course of the year and is lower in summer months than in winter months. These differences are due to systematic differences in electricity consumption not related to heating (e.g., lighting, seasonal changes in industry etc.). The slope of the fit (Fig. 8d) is steeper (more negative) in winter than in summer indicating that during warmer summer months the temperature variations influence the electricity consumption less than in colder seasons.

Because of these differences between calendar months we further scaled each calendar month separately to follow the same linear dependence on temperature as the January. To normalize the values of calendar month *k* we used equation

2 $$\begin{aligned} E'' = E' + (c_{\text {Jan}} - c_k) + (d_{\text {Jan}} - d_k)T, \end{aligned}$$

where $E''$ is the final scaled electricity consumption, $E'$ is the scaled electricity consumption obtained from Eq. (1), $c_{\text {Jan}}$ and $c_k$ are the intercepts and $d_{\text {Jan}}$ and $d_k$ the slopes of the linear fits (shown in Fig. 8c,d) for January month and calendar month *k*.

The plot in Fig. 8b shows the final normalized electricity consumption values as a function of temperature. The plot also indicates the linear fit

3 $$\begin{aligned} E''=7833.4 (\pm 9.7)~\text {GWh} - 114.28 (\pm 1.12)~\text {GWh}/^\circ \text {C} \times T, \end{aligned}$$

where $E''$ is the normalized electricity consumption and *T* is the monthly average temperature. The standard errors of the fit parameters have been indicated in parentheses in Eq. (3). The correlation corresponding to this linear fit is extremely high 0.98 ($p<10^{-279}$) and the standard deviation of the residuals is 172.16 GWh. We used this fit to reconstruct representative normalized electricity consumption values for years 1950–1989 with Finland’s average temperatures obtained from the ERA-5 re-analysis. We also computed the uncertainty range for the reconstructed values.

### Trend removal from the datasets

The normalization procedures outlined above remove the part of the long-term trends in electricity consumption, which are not associated to temperature variation. However, the monthly temperatures in Finland do exhibit a rising linear trend over 1950–2021, which is related to the global warming. In order to concentrate on inter-annual variations we subtract a linear trend from all variables, i.e., temperature, normalized electricity consumption and aa index. The linear trends are slightly different for different calendar months and are therefore estimated and subtracted separately for each calendar month.

### Regression and correlation analysis

For estimating the relationships between variables, we use the basic linear regression of form

4 $$\begin{aligned} y_i = a + bx_i + \epsilon _i , i = 1,2,...n, \end{aligned}$$

where $y_i$ is the response variable, $x_i$ is the explanatory variable, regression coefficients *a* and *b* are the intercept and slope of the fit, $\epsilon _i$ is the residual term and index *i* goes from 1 to *n* (number of data points).

As outlined above we use the linear regression model to reconstruct past normalized electricity consumption values with monthly temperatures. In the analysis we then compute correlation coefficients between the electricity consumption and temperature/aa index. In such calculations it is important to consider the statistical uncertainty of the reconstructed electricity consumption values in order not to overestimate the correlation or its statistical significance.

In those calculations where reconstructed electricity consumption values are involved, a Monte Carlo simulation is used to obtain a distribution of the correlation coefficients. The simulation has 10,000 repetitions and in each repetition new values for the reconstructed electricity consumption were generated from Eq. (3) for years 1950–1989 and the measured values for years 1990–2021 were appended thereafter. In each repetition and for each monthly value new values for the intercept, slope and the residual term were generated from their corresponding statistical distributions. The intercept and slope follow Student’s T-distribution with $n-2$ degrees of freedom and mean/stadard deviation indicated by the numerical values of Eq. (3). The residual term $\epsilon _i$ to be added was generated from a Gaussian distribution with zero mean and a standard deviation of 172.16 GWh. For each repetition of the Monte Carlo simulation we then calculated the correlation coefficient between the generated electricity consumption time series and either temperature or aa index as discussed in the analysis above. The final correlation was taken as the mean of the 10,000 repetitions.

For the correlation coefficients the p-values representing the statistical significance were in all cases calculated by a Monte Carlo simulation instead of the conventional method based on Student’s t-test. This was done because the data points in the time series are not fully independent due to inherent autocorrelation. Furthermore, when segregating the data by QBO phase there is the potential of introducing low frequency aliasing to the resampled data series, which could be incorrectly attributed, e.g., to influence of aa, which also naturally contains these low frequencies. To overcome these problems our Monte Carlo simulation runs 10,000 repetitions and in each repetition one of the compared data series (the electricity consumption data or temperature or aa data, depending on which variables are studied) as well as the QBO time series is randomly circularly shifted^45^, the data points are segregated according to the time-shifted QBO and the Pearson correlation coefficient is calculated. The p-value of the correlation is determined by computing the fraction of those repetitions, where the magnitude of the correlation coefficient is larger than the magnitude of the correlation obtained for the unshifted data calculated before.

### Calculation of geopotential patterns by maximum covariance analysis (MCA)

The maximum covariance analysis (MCA) was used to find patterns of 1000 hPa geopotential height that explain a maximum fraction of the covariance between the geopotential and aa index or Finland’s average temperature. In other words, when monthly geopotential fields are projected onto the patterns found by the MCA one obtains a time series of this projection, which possesses the maximum covariance between the aa index time series or Finland’s average temperature time series.

The MCA is calculated by first defining the covariance matrix of the two datasets as

5 $$\begin{aligned} C_{xy} = \frac{1}{n-1} X^T Y, \end{aligned}$$

where *n* is the number of points (time samples), *X* is the standardized time series of the aa index or Finland’s surface temperature and *Y* is the geopotential height data matrix, where the monthly geopotential height maps are reorganized as rows of the matrix. In this study we only include the data from the Northern Hemisphere between latitudes 20$\vphantom{0}^\circ$ N–90$\vphantom{0}^\circ$ N. Before calculating the covariance matrix *C* the matrix *Y* is centered by subtracting from each column the average value of the corresponding column. Thereafter, each column is weighted by the area of the corresponding latitude-longitude grid box on the spherical surface.

A singular value decomposition (SVD) of the covariance matrix is then computed as

6 $$\begin{aligned} C_{xy} = U \Sigma V^T. \end{aligned}$$

The vector *V* contains the patterns of maximal covariance with the vector *X*.

The found patterns are the scaled by dividing the pattern with the standard deviation of all the grid point values before plotting. We also determined the statistical significance of the patterns with a Monte Carlo simulation of 10,000 repetitions. In each repetition the aa index or Finland’s surface temperature time series were randomly circularly shifted in time. This procedure retains the autocorrelation structure of the time series, but breaks the temporal association of the two datasets. Then the maximum covariance pattern of geopotential height was calculated as outlined above either for all data points or for a randomly selected set with the same number of data points as in either the QBO-E or QBO-W phase (when determining the significance for the patterns in these two phases). The p-value for each grid point was then determined by computing the fraction of those repetitions where the value of the grid point differs from the median of all repetitions more than the value in the original pattern obtained above.

### Consent to participate

All authors consented to participate in this study.

## Data Availability

Data used in this research is freely available at following websites: https://cds.climate.copernicus.eu, Copernicus Climate Change Service (Climate Data Store) website for ECMWF’s ERA5 reanalysis data, http://isgi.unistra.fr, ISGI website for aa-index data, and https://energia.fi/en/newsroom/publications/monthly_electricity_statistics.html Finnish Energy (Energiateollisuus) website for the total electricity consumption data.

## Code availability

The MATLAB code and data files used to perform the analysis is available upon request to the corresponding authors.
