# Correction: Electricity consumption in Finland influenced by climate effects of energetic particle precipitation

**DOI:** 10.1038/s41598-026-49799-z

**Published:** 2026-05-06

**Authors:** Veera Juntunen, Timo Asikainen

**Affiliations:** Space Physics and Astronomy Research Unit, University of Oulu, Oulu, Finland

Correction to: *Scientific Reports* 10.1038/s41598-023-47605-8, published online 23 November 2023

In the original version of this article, Figure 6 contained an error in the scatter plot for the panel ‘QBO – W’, as there was duplication of some information found on the panel ‘QBO – E’ of the same Figure.

The incorrect version of Figure 6 and its legend appear below:

**Fig. 6:**
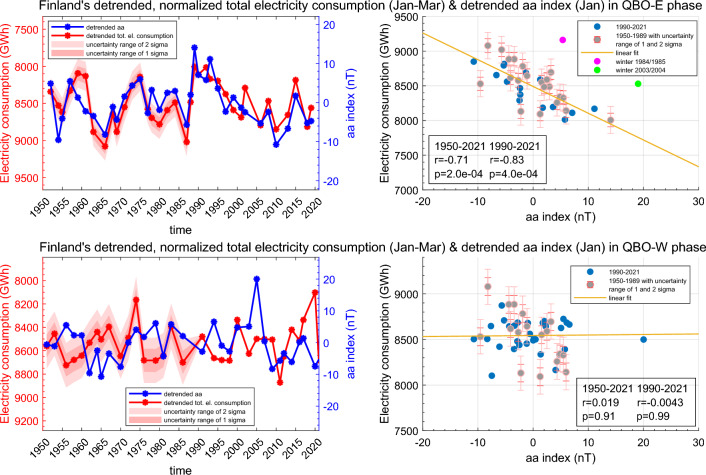
The normalized and detrended total electricity consumption (JFM average) and the detrended aa-index (Jan) during 1950–2021 for QBO-E winters (top row) and for QBO-W winters (bottom row). The QBO was taken from July preceding the winter season. The grey points in the scatter plots indicate reconstructed electricity consumption values. The error limits indicate an uncertainty range of 1 and 2 standard deviations (sigmas) for the reconstructed electricity consumption values. The correlation coefficients indicated in the scatter plots have been computed by excluding the two outlier winters of 1984/1985 and 2003/2004 (pink and green).

The original Article has been corrected.

